# JSAMS Plus starts publishing

**DOI:** 10.1016/j.jsampl.2022.100008

**Published:** 2022-11-01

**Authors:** Tim Meyer

**Affiliations:** Saarland University, Germany.

With the first articles of ***JSAMS Plus*** being published we have now visibly started a modern type of sport science journal with online only access, i. e. no print issues. Probably most readers will agree that in the foreseeable future this may become the regular publication model which will replace traditional scientific publishing with hard copies being stored in libraries. Already now the vast majority of readers from the scientific community rather download articles than consult complete magazines. In this sense, ***JSAMS Plus*** follows existing trends but at the same time wants to generate its own footprints, of course.

This can be facilitated by a proper choice of articles but shall be accompanied by innovative ways of article management in the future, too. Without revealing too many secrets, such procedural adaptations will have to take into account the specificities of a young online journal and the resulting expectations from authors (quick and correct publication) and readers (distinct journal profile meeting the current requirements of the sports medicine/science community). Such intentions may need some degree of flexibility that established journals like **JSAMS** do not necessarily have due to their more traditional structures among them the - well justified - extensive quality control systems. This means that ***JSAMS Plus*** has not been created as an online copy of JSAMS but as a distinct entity with its own profile which will become well visible in the near future.

**JSAMS**′ sister journal has been officially launched already in early 2022, and now the first articles are through its regular review system. This does not mean that JSAMS Plus is established but it has successfully made its first steps. And the first papers illustrate what the scope of ***JSAMS Plus*** will be. Besides three full-length articles:

• Smyth et al. - Injury surveillance at the 17/U & 19/U Australian National Netball Championships
• Abrahamson et al. - Cam morphology, hip range of motion and hip pain in young skiers and soccer players
• van Hooren et al. - Isometric single-joint rate of force development shows trivial to small associations with jumping rate of force development, jump height, and propulsive duration

there are three review papers:

• Gonçalves Gomide et al. - The role of physical activity in the clinical outcomes of people diagnosed with Covid-19: a systematic review
• Illmer et al. - The effects of weather factors and altitude on physical and technical performance in professional soccer: a systematic review
and one case study/short report:
• Silbernagel et al. - Markerless motion capture: What Clinician-Scientists need to know right now

This is complemented by an opinion paper from John Orchard: Knee Osteoarthritis in Australia: a 20-year case study of funding-system failure, medical waste and poor outcomes.

In terms of content, from sport injuries (Smyth et al., Abrahamson et al., Orchard) over a clinical topic from sport and exercise medicine (Gonçalves Gomide et al.) to biomechanical reports (van Hooren et al., Silbernagel et al.) - most relevant areas are covered or tackled. Hopefully, this meets your interests and you will accompany us through the future path of ***JSAMS Plus***.

From our end, we - the members of the editorial team - can promise that suggestions, comments and constructive criticism will always be appreciated and seriously taken into consideration. Looking forward to an interesting future with ***JSAMS Plus*** …

